# Levetiracetam treatment leads to functional recovery after thoracic or cervical injuries of the spinal cord

**DOI:** 10.1038/s41536-021-00121-7

**Published:** 2021-03-02

**Authors:** Rui Lima, Eduardo D. Gomes, Jorge R. Cibrão, Luís A. Rocha, Rita C. Assunção-Silva, Cláudia S. Rodrigues, Andreia Neves-Carvalho, Susana Monteiro, António J. Salgado, Nuno A. Silva

**Affiliations:** 1 Life and Health Sciences Research Institute (ICVS), School of Medicine, University of Minho, Braga, Portugal; 2 ICVS/3B’s-PT Government Associate Laboratory, Braga/Guimarães, Portugal

**Keywords:** Spinal cord injury, Regeneration and repair in the nervous system

## Abstract

Spinal cord injury (SCI) leads to dramatic impairments of motor, sensory, and autonomic functions of affected individuals. Following the primary injury, there is an increased release of glutamate that leads to excitotoxicity and further neuronal death. Therefore, modulating glutamate excitotoxicity seems to be a promising target to promote neuroprotection during the acute phase of the injury. In this study, we evaluated the therapeutic effect of a FDA approved antiepileptic drug (levetiracetam-LEV), known for binding to the synaptic vesicle protein SV2A in the brain and spinal cord. LEV therapy was tested in two models of SCI—one affecting the cervical and other the thoracic level of the spinal cord. The treatment was effective on both SCI models. Treated animals presented significant improvements on gross and fine motor functions. The histological assessment revealed a significant decrease of cavity size, as well as higher neuronal and oligodendrocyte survival on treated animals. Molecular analysis revealed that LEV acts by stabilizing the astrocytes allowing an effective uptake of the excess glutamate from the extracellular space. Overall, our results demonstrate that Levetiracetam may be a promising drug for acute management of SCI.

## Introduction

Spinal cord injury (SCI) leads to severe neurological deficits with a strong impact on the physiologic, psychological and social behavior of SCI individuals. Hence, it is urgent to develop therapeutic strategies that can specifically target this medical condition.

After the initial trauma that lacerates, compresses or contuses the spinal cord, a cascade of events is initiated, contributing to further tissue damage^1^. Within minutes following a SCI, the extracellular excitatory amino acid concentrations increase to neurotoxic levels, leading to excitotoxicity and neuronal cell death^2^. So, modulating the excitatory amino acid levels after trauma may be a valuable strategy to reduce acute neurotoxicity and protect the spinal cord. Therefore, in this work we explored the therapeutic action of a new antiepileptic drug —Levetiracetam (LEV)—known for binding to the synaptic vesicle protein SV2A in the brain and spinal cord and inhibiting presynaptic neurotransmitter release^3–7^.

As a matter of fact, LEV is able to increase the expression of glutamate transporters^8^ and prevent astrocytic dysfunction under inflammatory conditions^9,10^. These properties make LEV an interesting drug to modulate glutamate excitotoxicity in the acute phase of SCI. Thus, we explored the therapeutic value of LEV in two SCI models (thoracic and cervical), by assessing the impact of treatment at the behavioral, histological and molecular level and explored possible mechanisms underlying the effects of LEV.

## Results

### Acute LEV treatment promotes functional recovery in both thoracic and cervical injuries models

**Fig. 1:**
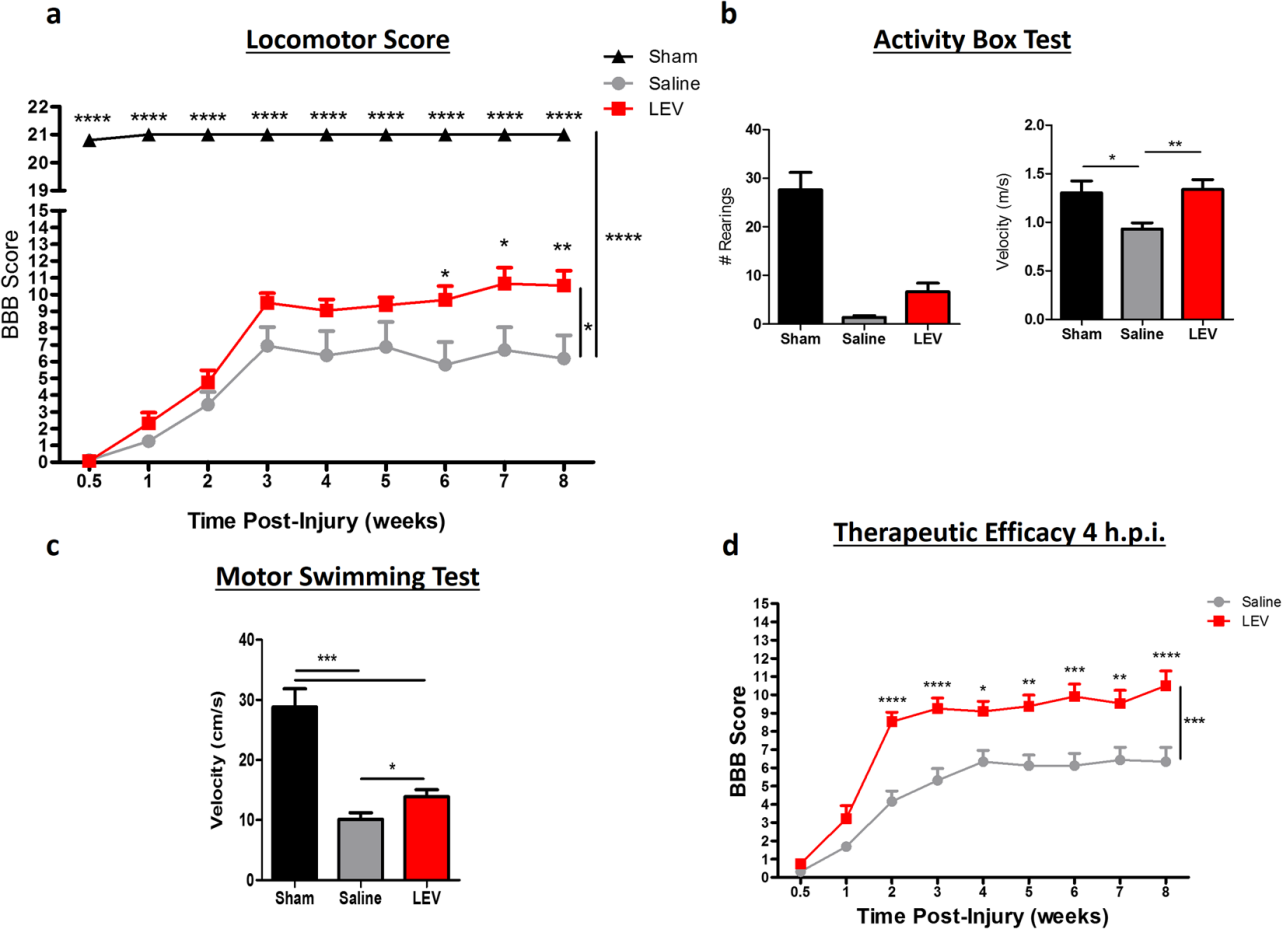
LEV treatment promotes significant functional locomotor recovery after thoracic SCI. **a** LEV-treated animals 8 weeks post injury were able to perform occasional weight-supported plantar steps, while saline-treated animals were only able to perform extensive movement of two joints and a slight movement of the third. Moreover, from 6th to 8th week post injury, the locomotor functions of LEV-treated rats were significantly higher than the saline-treated animals. **b** LEV-treated rats presented higher number of rearing and significantly higher velocity during locomotion. **c** Motor swimming test demonstrated that LEV treatment leads to significant better locomotor performance when compared to saline-treated animals. Sham *n* = 3; Saline *n* = 8; LEV *n* = 14. **d** LEV treatment 4 hpi is also able to promote significant functional locomotor recovery after SCI. From the 2nd to the 8th week post injury, the locomotor functions of LEV-treated rats were significantly higher than the saline-treated animals. Saline *n* = 15; LEV 4 hpi *n* = 15. Values shown as mean ± SEM. *p < 0.05; **p < 0.01; ***p < 0.001; ****p < 0.0001.

To assess the therapeutic potential of LEV, twenty two rats were subject to a thoracic contusion at T8 level and randomly distributed into two groups (Saline, *n* = 8; LEV, *n* = 14). Sham animals (*n* = 3) were only subjected to a laminectomy at T8. Functional recovery was analyzed using the Basso, Beattie and Bresnahan (BBB) score, Activity Box Test (ABT) and the Motor Swimming Test (MST). The BBB was performed weekly and revealed that although both LEV and saline-treated animals presented a spontaneous recovery over time, LEV-treated animals presented a significantly better functional recovery along 8 weeks when compared to saline treatment (Fig. 1a; *p* = 0.0223). LEV-treated rats presented at week 8 a mean BBB score of 10.5 ± 3.3, corresponding to the ability to support their own body weight and to perform occasional plantar steps, while saline-treated group presented a mean BBB score of 6.5 ± 3.5, which is translated into extensive movement of two joints and a slight movement of the third. We then assessed the therapeutic efficacy when LEV was administered 4 hpi in a set of 30 rats. LEV-treated animals presented, once again, a significantly better functional recovery along the 8 weeks when compared to saline-treated group (Fig. 1d; *p* = 0.0002), with a mean BBB score of 10.5 ± 3.1 comparing to 6.3 ± 3.0 for the saline-treated group. LEV-treated animals were able to perform occasional weight-supported plantar steps, while saline-treated animals were only able to perform extensive movement of two joints and a slight movement of the third.

Locomotor velocity and rearing activity was evaluated using the ABT at 8 weeks post injury. LEV-treated animals presented better locomotor abilities compared to saline-treated rats, both in the number of rearing movements and locomotor velocity (Fig. 1b); LEV presented a mean number of rearing movements of 6.6 ± 6.4 and a mean velocity of 1.34 ± 0.25 m/s, while the saline-treated group presented a mean number of rearing movements of 1.4 ± 1.1 and a mean of 0.93 ± 0.18 m/s velocity (*p* = 0.0042).

Locomotor recovery was further assessed by measuring swimming velocity. While LEV-treated rats presented an average velocity of 13.9 ± 2.6 cm/s, saline-treated animals presented an average swimming velocity of 10.1 ± 3.2 cm/s (Fig. 1c, *p* = 0.0458).

**Fig. 2:**
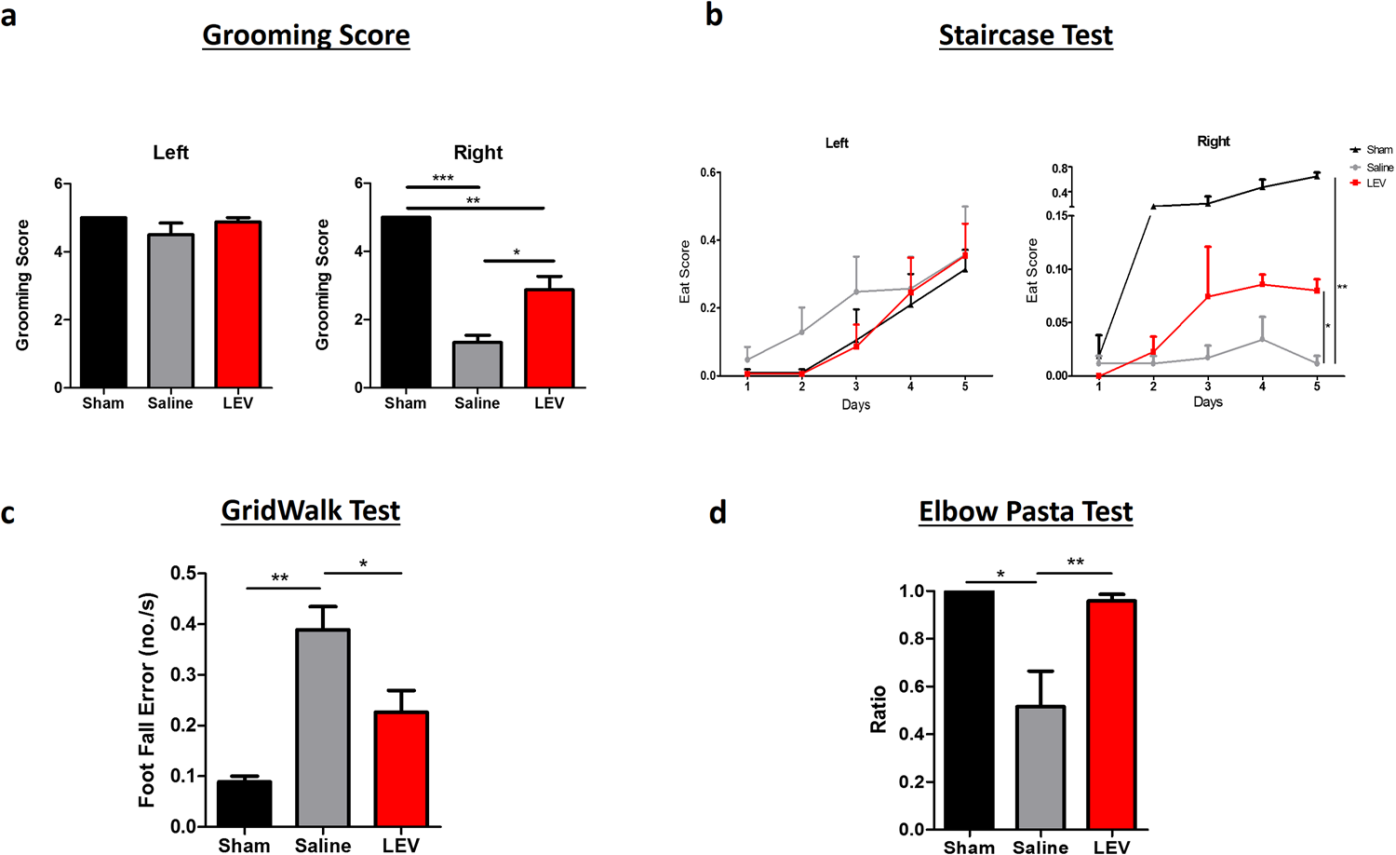
LEV treatment promotes functional recovery after cervical SCI. **a** Grooming score test demonstrated that LEV promotes a significant functional recovery of the right forelimb. LEV-treated animals were capable of reaching the eye level with the lesioned paw, while saline-treated animals were only capable of reaching underneath the snout. **b** The behavioral analysis of the staircase test revealed that LEV-treated animals were able to eat more sugar pellets with the right forepaw when compared to saline-treated rats. **c** The gridwalk test was also performed to complement fine locomotor recovery assessment. LEV treatment was able to significantly reduce the rate error of the animals while exploring the grid. **d** Finally, the elbow pasta test was also performed to assess bimanual dexterity of the rats. On this behavioral analysis, LEV-treated animals demonstrated higher motor abilities being able to eat the pasta with both forepaws for longer time periods than the saline treatment rats. Sham *n* = 3; Saline *n* = 6; LEV *n* = 8. Values shown as mean ± SEM. *p < 0.05; **p < 0.01; ***p < 0.001.

Following the assessment of hindlimb recovery associated with SCI in rats, we also explored the LEV treatment impact on the forelimb behavior of injured rats at the cervical level. For this purpose, fourteen rats were subjected to a cervical right hemisection at C2 and were randomly divided into two treatment groups (Saline, *n* = 6; LEV, *n* = 8). Once again Sham animals (*n* = 3) were only subjected to a laminectomy at C2. The behavioral recovery analysis of cervical SCI rats was assessed using behavioral tests that are highly dependent on supraspinal activation controlling voluntary movements. Eight weeks post injury, LEV-treated rats presented a grooming score of 2.9 ± 1.1 for the right forelimb (injury side), which represents a significant higher score (*p* = 0.0093) when compared to the saline group that presented a grooming score of 1.3 ± 0.8 (Fig. 2a). These grooming scores mean that LEV-treated animals were able to reach the eye level during grooming, while the saline-treated animals were only able to reach the underneath of the snout. On both LEV and saline-treated animals, the left forelimb was barely affected by the right hemisection, presenting scores very close to 5 (Fig. 2a).

The staircase test was used to assess side-specific deficits in paw reaching and grasping. While no significant differences between groups in level and touch scores were found, the ‘eat score’ for the right forepaw was significantly different (Fig. 2b; *p* = 0.0306), with the saline-treated animals presenting a lower ‘eat score’ (0.012 ± 0.015) comparing to the LEV-treated animals (0.080 ± 0.024). Importantly, although all animals were able to reach the same level on the staircase and touch the same number of pellets, LEV-treated animals were able to hold the pellets for longer periods, and thus ate more pellets.

In order to complement the functional recovery analysis, animals were also subjected to the gridwalk test. LEV treatment promoted a significant reduction (*p* = 0.0499) of foot fall errors per second (0.25 ± 0.10) compared with saline treatment (0.39 ± 0.11 errors per second) (Fig. 2c), evidencing once again its therapeutic value.

Finally, forepaws functionality was evaluated using the elbow pasta test (EPT). LEV-treated animals presented a ratio of 0.96 ± 0.07, while the saline-treated animals presented a ratio of 0.52 ± 0.33 (*p* = 0.0059). This shows that animals treated with LEV handled the pasta significantly more time with both paws (around 96% of the time) than the animals treated with saline (around 52% of the time).

### LEV acute treatment reduces lipid peroxidation and modulates glutamate excitotoxicity

**Fig. 3:**
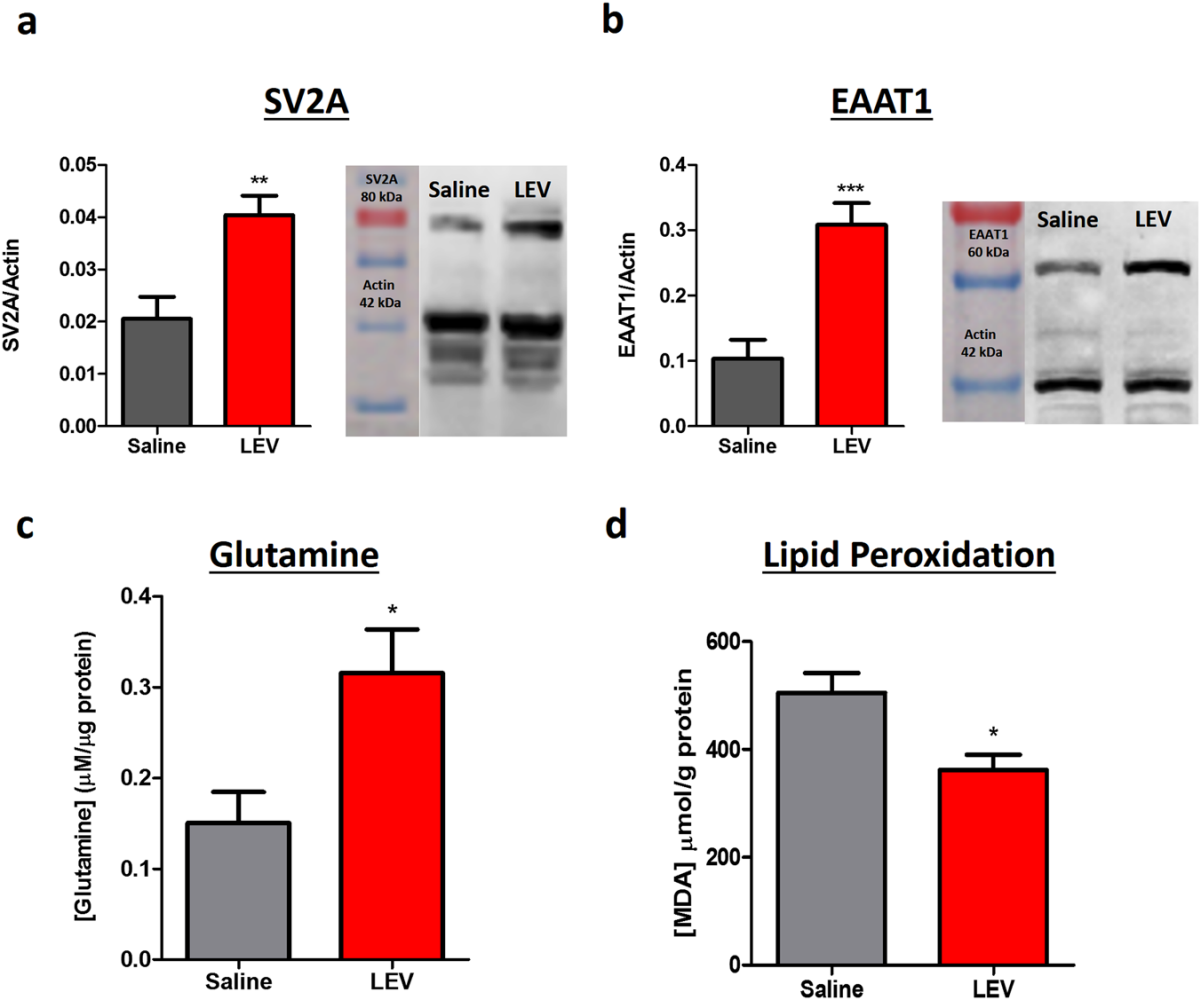
LEV treatment promotes neuroprotection by modulating glutamate excitotoxicity and reducing lipid peroxidation. **a** LEV treatment led to a statistical significant increase of SV2A on the spinal cord tissue. **b** LEV also promoted a significant increase of EAAT1 on the spinal cord tissue. **c** LEV treatment increased glutamine tissue concentration 24 h post injury. **d** LEV promoted a significant reduction of lipid peroxidation on spinal cord tissue 24 h post injury. Saline *n* = 6; LEV n = 6. Values shown as mean ± SEM. *p < 0.05; **p < 0.01; ***p < 0.001.

One hour post injury (hpi) western blot (WB) analysis revealed a significant increase of the synapse vesicle glycoprotein SV2A in LEV-treated animals (Fig. 3a; *p* = 0.0051). LEV treatment restored the amount of the SV2A to healthy control values (Supplementary Fig. 1a). WB also shown a significantly higher concentration of the excitatory amino acid transporter 1 (EAAT1) on LEV-treated animals when compared to saline treatment (Fig. 3b; *p* = 0.0003). However, the increase of EAAT1 was not observed on the spinal cord tissue of healthy animals treated with LEV (Supplementary Fig. 1b), meaning that this increase is potentiated by LEV only on pathological conditions. In order to dissect the effect of LEV on glutamate reuptake, primary cultures of astrocytes (Supplementary Fig. 1c) were incubated with 1 mM of glutamate and its concentration on the culture medium was measure by high-performance liquid chromatography (HPLC). Results revealed that astrocytes incubated with LEV were able to significantly uptake more glutamate from the culture medium than astrocytes without treatment (Supplementary Fig. 1d; *p* = 0.0084). Twenty-four hours post injury, the glutamine concentration on the spinal cord tissue was determined by HPLC. The LEV-treated group presented higher concentration of glutamine when compared (*p* = 0.0486) to the saline-treated group (Fig. 3c).

The levels of lipid peroxidation of the spinal cord tissue were assessed by the Thiobarbituric acid reactive substances (TBARS) method^11^. LEV-treated animals presented significant lower lipid peroxidation when compared to the control group (Fig. 3d; *p* = 0.0355). Serum from blood samples was collected 1 and 24 hpi and cytokines quantification was performed by multiplex analysis. The levels of interleukin (IL)-1β, IL-6, IL-10, interferon (IFNγ), tumor necrosis factor alpha (TNFα), and IL-4 were analyzed, however, no differences were found between groups on both time points (Supplementary Fig. 2). Of note, sham presented similar cytokines levels as the SCI animals due to the tissue damage that these rats suffered on the muscles and bone in order to create a laminectomy. Finally, the effect of LEV treatment on the immune response after SCI was also analyzed by flow cytometry. Seven days post injury, the spinal cord tissue was collected and the concentration of different leukocytes’ populations (microglia, infiltrating lymphoid and myeloid cells) was evaluated. Analysis did not revealed any significant effect of LEV on these cellular populations (Supplementary Fig. 3).

### LEV treatment increases motor neuron and oligodendrocytes survival, neuronal fiber preservation, and reduces cavity size and microglia reactivity

**Fig. 4:**
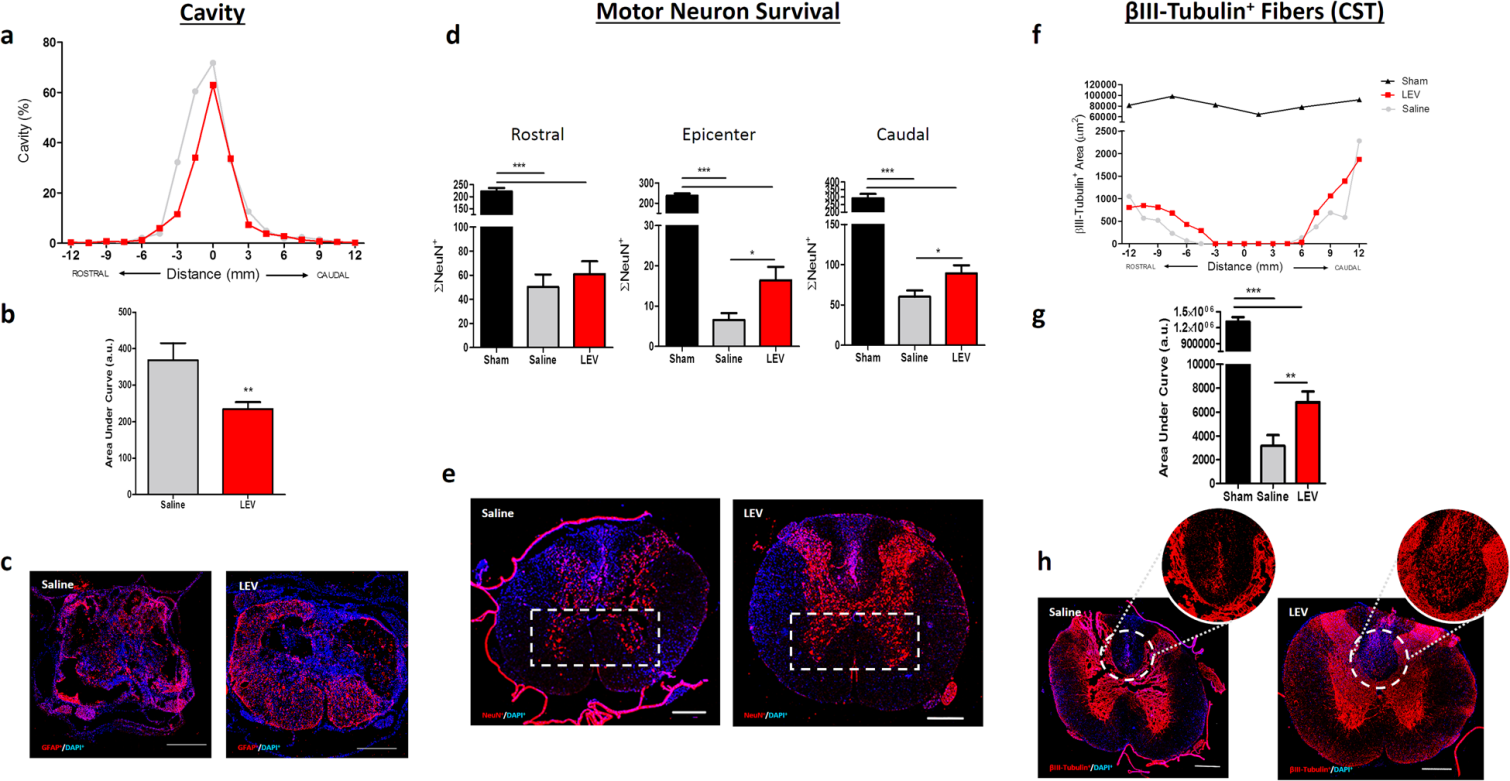
The effect of LEV on cavity size, motor neurons survival, and CST axons preservation. **a** Mean cavity size distribution along the rostral-caudal axis of LEV and saline-treated animals, **b** Cavity quantification revealed that LEV treatment significantly reduced cavity size after SCI. Sham animals did not presented any cavitation. **c** GFAP + staining was used to demarcate the cavity of both LEV and saline-treated animals. **d** The sum of motor neurons was determined at rostral, caudal and epicenter regions. LEV treatment was able to significantly protect motor neurons at epicenter and caudal regions. **e** Representative confocal photomicrographs of NeuN+ cells. **f** Rostral-caudal distribution of positive neuronal fiber staining at CST. **g** Quantification of βIII-Tubulin+ staining at CST revealed significant neuronal fiber preservation on LEV-treated rats. **h** Representative confocal photomicrographs of βIII-Tubulin+ staining. Values shown as mean ± SEM. *p < 0.05; **p < 0.01; ***p < 0.001. Scale bar = 400 µm. Sham *n* = 3; Saline *n* = 8; LEV *n* = 14.

Histological analysis was performed at the chronic phase (8 weeks post injury) in both thoracic and cervical injuries. The lesion size was quantified in the T8 contusion animal set using GFAP^+^ staining. The rostral-caudal analysis of spinal cord tissue revealed that LEV treatment promoted a reduction of cavity size, a ~36% reduction (100 − [(AUC_LEV_/AUC_Sal_)*100]) in lesion size when compared to saline-treated group. This reduction was more accentuated on the rostral part of the spinal cord (Fig. 4a, b, *p* = 0.0045).

Subsequently, we explored the effect of LEV treatment on specific tracts and cell populations of the spinal cord. Motor neurons death is a characteristic hallmark of SCI and contributes to permanent functional deficits. Hence, the effect of LEV treatment on motor neurons survival was assessed. The neuronal marker NeuN was used to stain neurons and positive cells were quantified on the ventral horns along the rostral-caudal axis (Fig. 4d). As shown in Fig. 4d, LEV treatment promoted a marked increase in motor neurons survival, namely at the epicenter (*p* = 0.0291) and caudally to the epicenter (*p* = 0.0469).

Additionally, we also evaluated axonal preservation of the corticospinal tract (CST) using βIII-tubulin^+^ staining along the rostral-caudal axis (Fig. 4f). The quantification of βIII-tubulin^+^ staining revealed a significant preservation (Fig. 4g; *p* = 0.0255) of CST neuronal fibers in LEV-treated animals. Sham histological representation of motor neurons and βIII-tubulin+ staining on the CST can be found in supplementary data (Supplementary Fig. 4).

The βIII-tubulin^+^ staining were also used to assess axonal preservation after a hemisection on the cervical injured animal set. Once again, the quantification of neuronal fibers revealed higher positive staining on LEV-treated animals when compared with the control group at rostral (*p* = 0.004), caudal (*p* = 0.0381) and epicenter (*p* = 0.0323) areas of the cervical spinal cord (Supplementary Fig. 5a, b), supporting the neuroprotective effect of LEV treatment. Oligodendrocytes, a highly vulnerable cell population to glutamate excitotoxicity^12^, were also protected by LEV treatment (Supplementary Fig. 5c, d; *p* = 0.0187). Finally, positive stained area for reactive resident microglia and infiltrated macrophages were reduced by LEV treatment both on thoracic (Supplementary Fig. 6a, b; *p* = 0.0118) and cervical injured animals (Supplementary Fig. 6c; *p* = 0.037).

## Discussion

It is well described that excitotoxicity plays a central role in the secondary damage resulting from a CNS injury^13^. Overactivation of glutamate receptors results in accumulation of intracellular calcium and sodium, producing cytotoxic edema and intracellular acidosis. Excitotoxicity initiates a cascade of events that culminates with the activation of calcium-related proteases, nitric oxide synthase stimulation, production of free radicals, lipid peroxidation, enzymes inactivation of glyceraldehyde-3-phosphate dehydrogenase, and other oxidative processes^14,15^. These events result in neurodegeneration and neuronal death, pointing out the importance of modulating glutamate excitotoxicity after SCI. Despite the extensive search for the mechanisms of excitotoxicity, however, there are currently no pharmacological interventions in the clinical setting capable of providing effective and sustained neuroprotection.

In this study, we propose the use of Levetiracetam as a neuroprotective drug for SCI acute treatment. It was demonstrated that LEV has a unique mechanism of action and a novel binding site (SV2A) that leads to the decrease of neurotransmitters release^3^. Additionally, LEV presents excellent pharmacokinetic properties such as high solubility in water, rapid absorption and distribution, linear kinetics, good bioavailability and low toxicity^16–18^.

Having this in consideration, we tested LEV neuroprotective effect after traumatic SCI. Using a contusion injury model at thoracic level (T8) we observed that LEV treatment promoted a marked functional recovery. When treated with LEV, SCI rats were able to achieve weight supported plantar steps, while control animals were only able to perform extensive movements of two joints and a slightly movement of a third joint. This improvement on the hindlimb motor skills is probably the reason why the treated animals also walked over ground and swam faster than non-treated animals. These results suggest that LEV treatment has a beneficial effect on incomplete paraplegia. However, the BBB scale may not give a valuable and precise information about cortical-dependent movement^19^. Rating scales provide information on the activation of spinal networks that are able to produce a coordinated stepping pattern^20,21^. In rodents, these pattern generating networks are activated by the ventrally located reticulospinal tract^22^. Consequently, we also evaluated if the therapeutic effects of LEV were maintained using behavioral tests highly dependent on cortical activation. In order to assess this, we used a cervical hemisection injury (C2). Animals were treated with the same dosage and the motor skills of the forelimbs were evaluated using four behavioral tests. The animals were subjected to Gridwalk test, Elbow Pasta test, Staircase test and the Grooming score, and the LEV-treated animals significantly outperform the saline-treated animals in all of them. These behavioral tests explore fine motor skills recovery and are highly dependent on supraspinal activation. For instance, in the gridwalk test animals need to accurately place the limbs on a wire mesh. This requires forelimb/hindlimb coordination, which is mediated by ventrolateral tracts^23^, a functioning reticulospinal system to initiate the stepping rhythm, as well as voluntary movement control, which is predominantly mediated by the corticospinal tract^24,25^. During the staircase test or the elbow pasta test, animals need good dexterity to reach and grab the food pellets or handle the pasta, and these behaviors are dependent of cortical activation^26,27^. Lastly, grooming behavior is dependent of dorsolateral and ventromedial neostriatum neuronal activation^28^. As we observed a beneficial effect of LEV in these parameters, we suggest that it may also have a great therapeutic potential for cervical injuries. Admittedly, incomplete tetraplegia is the most common type of injury in humans, characterizing around 48% of the SCI clinical cases^29^, which denotes the clinical relevance of this findings.

In order to understand the underlying mechanism for the effect of LEV on functional recovery, we performed molecular and histological analysis. We found that lipid peroxidation was reduced in LEV-treated animals. Lipid peroxidation is triggered when highly reactive oxygen radicals react with membrane polyunsaturated fatty acids, resulting in disruption of membrane integrity and compromising cell function, which may lead to cell death. Glutamate receptors activation leads to an elevation of intracellular calcium that induces the formation of free radicals and thus lipid peroxidation. It has been demonstrated that LEV modulates the intracellular calcium levels either by inhibiting neuronal high-voltage-activated calcium channels^30^ or by reducing calcium release from endoplasmic reticulum^31^. The ability of LEV to modulate the two major pathways, leading to intracellular calcium increase demonstrated an important molecular effect of this agent in neurons and may explain the neuroprotective role herein observed. Admittedly, we observed that LEV treatment significantly increased the synaptic vesicle glycoprotein SVA2 when compared to saline treatment alone. The detailed role of SVA2 in regulating synaptic neurotransmitter release remain to be clarified, however, it is known that SV2A KO mice developed severe seizures and die within weeks^6,32^, suggesting that the absence of SV2A may be correlated with the presence of high levels of excitatory amino acids. So, the restoration of SV2A values after SCI by LEV probably led to a modulation of glutamate release. However, our molecular analysis also revealed significant higher glutamine levels in the spinal cord tissue of the animals treated with LEV. Glutamate released by neurons is uptaken by astrocytes and then converted in glutamine by glutamine synthetases. These results indicated that LEV may be acting in a different manner than just inhibiting pre-synaptic glutamate release. It has been demonstrated that astrocytes cultured under pro-inflammatory conditions lose some regulatory properties, namely ion and neurotransmitter uptake capabilities^9^. Moreover, co-culturing astrocytes with activated pro-inflammatory microglia leads to a loss of astrocytic gap junction coupling^33^. Figiel et al., demonstrated that the coupling capacity of astrocytes is directly linked with glutamate uptake, and decoupling of astrocytes resulted in decreased expression of astrocytic glutamate transporter and a 60% reduction in glutamate uptake^33^. In addition, Haghikia et al.^9^ have shown that LEV prevents impairment of astroglial regulatory properties under inflammatory conditions. Taking this into account, we analyzed the effect of LEV on astrocytes after a SCI. We found higher amounts of the EAAT1 glutamate transporter in the spinal cord tissue of LEV-treated animals. This glutamate transporter is mainly expressed in astrocytes, and it is responsible for the glutamate reuptake from the extracellular space^34^. The increase of EAAT1 explains the rise of glutamine levels, accordingly to the previous observations, indicating that LEV is acting through the stabilization of astrocytes and promoting glutamate uptake. In order to test this hypothesis, we performed an in vitro assay where glutamate was added to primary cultures of astrocytes. Glutamate concentration in the culture medium was then measured by HPLC. Astrocytes incubated with LEV were able to significantly uptake more glutamate than astrocytes without treatment. This result supports that LEV is able to reduce excitotoxicity by acting on astrocytes.

Next, we analyze the long-term effects of LEV treatment at histological level. Histological analysis of thoracic injured animals demonstrated that LEV treatment significantly reduced the cavity, suggesting a sustained protective effect of the spinal cord tissue. The analysis on specific areas and populations further revealed that treatment significantly protected the motor neurons on the ventral horns and the axons of the corticospinal tract. On cervical injuries, it was also possible to observe a neuroprotective effect of LEV on neuronal fibers as well as an increased survival of oligodendrocytes. The reduction of excitotoxicity pathways by LEV promoted the survival of important neuronal populations (motor neurons and oligodendrocytes) and crucial fibers tracts. The neuronal protection observed supports the significant functional recovery promoted by LEV. Furthermore, the histological analysis also revealed that the immune response on the spinal cord tissue was significantly reduced with LEV treatment. However, molecular and cellular analysis performed by multiplex and flow cytometry did not reveal any difference on the immune response between treated and non-treated animals, which suggests that the reduction on the staining for microglia/macrophages is probably due to the lower extension of neurodegeneration than to a direct effect on inflammation by LEV.

Notably, we found that LEV was able to promote functional recovery when administered 4 hpi at similar level as compared to administration immediately after injury. This scenario better mimics what usually happens in clinic and shows the clinical relevance of LEV treatment. Nonetheless, a 4 h waiting period is still not optimal from a translational perspective, so our future research will be focused on determining the complete time window of efficacy of LEV.

Wang and colleagues^35^ pioneered the demonstration that LEV has a neuroprotective action after a traumatic brain injury (TBI). The authors demonstrated that a single administration of LEV was able to reduce neuronal death and improve motor behavior. This result was later replicated by the OBTT consortium, strengthening the evidences of the neuroprotection action of LEV treatment after TBI. OBTT showed that LEV had great beneficial effects in two TBI models across multiple outcomes, including motor, cognitive, and histology^36^. Recently, Nursoy et al. observed that the administration of LEV 4 hpi is able to protect the spinal cord tissue in the acute phase (by reducing bleeding severity, vacuolization and necrotic neuron count results.). The neuroprotection observed was slightly higher than with methylprednisolone administration^37^. However, the authors reported that the effects could not be sustained in the subacute period. Moreover, it was reported that LEV does not seem to be biochemically effective in reducing or stopping the inflammatory processes. Likewise, we also did not observe a direct impact of LEV on the immune response. Nevertheless, our analysis focus was not only on the acute/sub-acute phase, but also on the long-term effects. We observed that the acute neuroprotection has a positive impact on the chronic phase, both histologically and functionally.

Overall, we demonstrated that LEV is a promising therapeutic drug for SCI acute management. Our results strongly show beneficial effects of LEV treatment on both functional recovery and neural preservation. Considering the therapeutic effect of LEV, the clinical relevance of our findings, the translational potential of this therapeutic strategy and LEV’s track record for safety, its favorable pharmacokinetics and the absence of detrimental interactions with other drugs^38^, we consider that LEV has the potential to be applied to SCI clinical practice.

## Methods

### Study design

The objective of this study was to evaluate the therapeutic potential of LEV treatment after SCI. We accessed LEV treatment effects on paraplegia and tetraplegia models, evaluating functional recovery, histological and molecular alterations.

The number of animals on this study was determined by Gpower software (University of Kiel, Germany) with results obtained from a pilot study with *n* = 5 animals per group. Animals were randomly treated with LEV or Saline and all data collection (behavior and histology) was obtained blindly to the treatment group. All procedures were carried out in accordance to EU directive 2010/63/EU and were approved by the ethical committee in life and health sciences (ID: SECVS116/2016, University of Minho, Braga, Portugal).

During data analysis, an exclusion criteria was established for both behavioral and histological assessments. Animals that did not present a BBB of zero three days after injury were excluded of the behavioral and histological analysis. Spinal cord sections that were shattered, cracked, folded or sections washed off during immunostaining procedure were excluded from analysis. All procedures were replicated twice.

### Spinal cord injury models and treatment

This study has used one hundred and fourteen Wistar Han female rats (10 weeks old, weighting 190–260 g). Animals were kept under standard laboratory conditions (12 h light: 12 h dark cycles, 22 °C, relative humidity of 55%, ad libitum access to standard food and water), and paired housed. Animal handling was carried out 3 days prior to surgery.

General anesthesia was induced by an intraperitoneal injection (i.p.) of ketamine (100 mg/ml, Imalgene/Merial, Georgia, USA) and medetomidine hydrochloride (1 mg/ml, Dormitor/Pfizer, New York, USA) mixture, at a volume ratio of 1.5:1. The fur was shaved from the surgical site and the skin disinfected with ethanol 70% and chlorohexidine. Surgical procedures were performed under sterile conditions. On this study, we evaluated the potential of LEV as a neuroprotective agent on two SCI rat models: a thoracic contusion model and a cervical hemisection model.

### Contusion model

Animals were subjected to a severe contusive SCI as previously described^39–41^. Briefly, the animals were placed in a prone position and a dorsal midline incision was made at the level of thoracic spine (T5–T12). The paravertebral muscles were retracted and the spinous processes and laminar arc of T8 were removed, and the spinal cord exposed. The dura was left intact. A weight drop trauma model that consisted in dropping a 10 g weight rod from a 10 cm height onto the exposed spinal cord was used. The rod was guided through a stabilized tube that was positioned perpendicularly to the center of the spinal cord.

### Cervical hemisection model

The procedure for cervical hemisection model was performed as described by Urban et al.^42^. Briefly, a 5 cm midline incision was made in the dorsal surface of the skin and muscle, then retractors were used to expose the surface of the C2 and C3 vertebrae. The spinous processes and laminar arc of C2 was removed, and the spinal cord exposed. The hemisection lesion was performed on the right side of the cord, with a perpendicular incision at midline using a sterile #11 surgical blade (Razormed, Haryana, India) at C2 level.

After the trauma, the muscles were sutured with Surgiquick suture (Sutures Ltd, Wales, United Kingdom) and the incision closed with surgical staples (Fine Science Tools, Heidelberg, Germany). Anesthesia was reversed using atipamezole (5 mg/ml, Antisedan/Pfizer). After injury, animals were randomly divided into two treatment groups: LEV (Levetiracetam (Kemprotec, UK) (750 mg/kg) and saline treatment. Sham animals were not subjected to a SCI being exposed only to a laminectomy. Treatment was administrated by intraperitoneal injection immediately after injury.

Post operative care for all rats included butorphanol (Richter Pharma AG, Wels, Austria) administration twice a day, for a five-day period as well as vitamins (Duphalyte, Pfizer, USA), saline, and enrofloxacin (Bayer, Leverkusen, Germany), twice a day for a 7-day period. Manual expression of bladders was performed twice a day until animals recovered spontaneous voiding. Body weight was monitored weekly as a parameter of general health of the animals. If a weight loss over 10% of body weight was detected, a daily high-calorie oral supplement (Nutri-Cal®) was administered. A weight body loss of more than 15% and/or presence of authophagy were defined as humane endpoints during post operative recovery.

### Molecular analysis

In order to better understand the mechanisms underlying LEV therapeutic effects, a molecular analysis was performed on the thoracic contusion rat model.

### Spinal cord tissue homogenate preparation

An one centimeter segment of spinal cord centered at epicenter was homogenized in cold RIPA buffer (50 mM Tris-HCl, 150 mM NaCl, 0,1% SDS, 1% NP-40, PMSF and a protease inhibitors cocktail (Complete; Roche, Switzerland)). Samples were sonicated for 5 min and centrifuged at 13,500 rpm for 5 min at 4 °C, supernatant was collected and stored at −80 °C. Protein concentration was determined using Bradford assay (BioRad,USA)^43^.

### Western blot

Samples were boiled at 100 °C for 5 min with laemmli buffer and β-mercaptoethanol. Fifty micrograms of total protein were loaded into 15% sodium dodecyl sulfate polyacrylamide gel electrophoresis gels and then transferred to nitrocellulose membranes. After overnight incubation at 4 °C with primary antibodies: rabbit anti-EAAT1 (1:500, Millipore, USA), rabbit anti-SV2A (1:500, Abcam, United Kingdom) and mouse anti-α-actin (1:1000, Abcam, United Kingdom), the secondary antibodies were incubated 1 h at room temperature following dilutions: anti-rabbit (1:10.000, BioRad, USA) and anti-mouse (1:10.000, BioRad, USA). Antibody binding was assessed by chemiluminescence (ECL kit, BioRad, USA). Band quantification was performed using Image-Lab software (BioRad, USA) according to the manufacturer’s instructions using α-actin as the loading control. All blots or gels derived from the same experiment and were processed in parallel.

### Lipid peroxidation

Spinal cord tissue homogenates were assayed for thiobarbituric acid reactive substances (TBARS), as an index of lipid peroxidation^11^. One hundred microlitersof spinal cord homogenate was added 1 ml of 50% trichloroacetic acid (TCA) and 2.7% of thiobarbituric acid (TBA) and incubated for 1 h at 100 °C in water bath. After incubation, the tubes were put on ice until they were ice cold (~5 min). The TBA precipitate was pelleted by centrifugation for 5 min at 13,500 rpm (4 °C). The supernatant was transferred to the 96-well plate and absorbance read at 532 nm subsequent to subtraction of non-specific absorption at 600 nm (Infinite® M200 NanoQuant microplate reader, Tecan, Switzerland). A standard curve for MDA concentration was performed and used the linear regression for determination of MDA in spinal cord tissue homogenate samples. The MDA concentration was normalized to protein content.

### Serum cytokine analysis

After 1 h and 24 h post injury, blood was collected from the tail and allowed to clot for 30 min before centrifugation (10 min at 10,000 × *g*). Then, serum was collected and frozen at −80 °C. An enzyme-linked immunosorbent assay for IL-1β, IL-8, IL-10, IFNγ, TNFα, and IL-4 detection (Millipore, USA) was used and the assay was performed as instructed by the supplier. Samples were analyzed in a MAGPIX Luminex’s xMAP® instrument (Luminex, Austin, TX, USA).

### High-performance liquid chromatography

The quantification of amino acid concentrations in spinal cord tissue homogenate was performed by HPLC. Briefly, spinal cord homogenate was filtered through 200 μm filters and diluted at 1:10 ratio. Two-hundred and forty microliters of diluted sample were added to HPLC tubes. All mobile phases (polar and apolar) for elution were filtrated and degasified at least for 45 min. The mobile polar phase was composed by disodium phosphate, propionic acid and acetonitrile; and the mobile apolar phase by acetonitrile and methanol. A precolumn derivatization method using ortho-phthalaldehyde 1:5 [(OPA) with methanol ≥99.9%, potassium borate 1 M pH 9.5, and 2-β-mercaptoethanol ≥99.0% (Sigma-Aldrich, USA)] to detect amino acids in a Gilson UV/vis_155 detector (338 nm) was used. Moreover, a Gilson pump system (Gilson) was used with a 40 °C Hi-Chrom C18 (model HI-5C18-250A) 5 μm particles column (Hi-Chrom) (Thermo Fisher Scientific, USA). All data was analyzed using the Gilson Uniprot Software version 5.11. Standard solutions of each amino acid were prepared in MilliQ water (Millipore, USA): l-aspartate, l-glutamate, l-asparagine, Histidine, l-serine, l-glutamine, l-arginine, Glycine, l-alanine, l-lysine, l-isoleucine, l-phenylalanine, l-methionine, l-tryptophan, l-cistine, and l-leucine (all from Sigma-Aldrich, USA).

### Behavioral assessment

Basso, Beattie, and Bresnahan (BBB) Score —The BBB locomotor rating scale^44^ was used to evaluate functional recovery. Researchers performed all behavioral tests blindly to the treatment groups. The BBB test was performed 3 days post injury and thereafter weekly for an 8-week period. A BBB score of 0 indicates no hindlimb movement. A BBB score of 1 through 8 indicates joint movement, but no weight support. A BBB score of 9 through 20 indicates an ability to support weight and use the limb for locomotion but with some degree of abnormality. A BBB score of 21 corresponds to the locomotion of a normal rat.

#### Activity box test (ABT)

This test allows the assessment of exploratory behavior by measuring the total distance traveled, velocity and number of rearing movements by the animals. The test was performed in an open arena (43.2 × 43.2 cm) with transparent acrylic walls (MedAssociates Inc., Vermont, USA). Animals started the test at the arena’s center and were given 5 min to explore it. Data was collected using the activity monitor software. Velocity and number of rearing movements was used as a measure of locomotor activity^45^.

#### Grid walk test (GW)

The GW test assesses deficits in descending motor control by examining the ability of the rat to navigate across 1.3 m long runway with regularly spaced gaps (5 cm) between round metal bars^46^. A training section was performed before surgery, where groups of 4 animals freely explored the grid for 10 min. The test consisted on recording each rat performance of a 3 min trial, where the animals freely explored the grid. For each 3 min trial, we selected 30 s based on continuous movements of the animal on the grid, and then determined the number of errors committed by the rat. For this analysis, the number of errors per second (n°/s) was calculated by dividing the total number of errors by the performance time (30 s).

#### Motor swimming test (MST)

Swimming is a natural behavior of rats and the buoyancy provided by water enables them to perform locomotor movements without having to totally support their body weight. After a short training period, the animals swim without any apparent aversion and the test is hardly influenced by symptoms of stress. The motor swimming test was performed on a quadrangular pool (water temperature 24–25 °C), where the rat had to reach the platform to get out of the water. Two days before the test, the animals performed four trials as training period. On the day of behavior analysis, all the trials were recorded by a videocamera. Velocity was assessed by using 3 trials of each rat. The software Ethovision XT 12 (Noldus, Wageningen, Netherlands) was used to determine the swimming velocity.

#### Grooming score (GS)

The forelimbs functionality was assessed by GS, using a scoring system originally developed to examine recovery in a rat brachial plexus reconstruction model^47^. Cool tap water was applied to the animal’s head and neck with soft gauze and the animal was placed in individual glass cylinders (50 cm height x 20 cm diameter). Grooming activity was recorded with a video camera from the onset of grooming through at least two stereotypical grooming sequences, which include (1) licking of the forepaws and face washing, (2) forelimb grooming of the face, (3) repetitive licking of the body, and (4) hindpaw scratching^48^. Slow motion video playback was used to score each forelimb independently by the maximal contact made while initiating any part of the grooming sequence. The grooming score was assigned depending on the highest region touched by the hand as follows: 0—no contact with the head; 1—contact with the mouth only; 2—contact with the snout bellow the eyes; 3—contact with the face from the eye level to below the ears; 4—contact with the ears; 5—contact with the head behind the ears.

#### Elbow pasta test (EPT)

The EPT consisted of two trials in 2 consecutive days with elbow pasta pieces given one at a time per each trial. Rats were placed in individual glass cylinders (50 cm height x 20 cm diameter) for the testing period. All rats were accustomed to eating pasta in the presence of the experimenter prior to testing. The trials were recorded with a video camera. As Whishaw et al.^27^ described, when eating pieces of pasta, healthy rats most often hold the piece in both paws. On this behavior assessment, we quantified the ratio between the time used by the animal to eat the elbow pasta with both paws by the total time needed to eat all the pasta. A mean was calculated between both trials.

#### Staircase test (ST)

The ST was performed in order to assess skilled forelimb motor function. ST test was executed as described by Carvalho et al.^49^. Briefly, the animals were trained prior injury, presented with the food pellets in their home cage and subsequently trained daily for five consecutive days on the ST apparatus (Model 80300, Campden Instruments, United Kingdom). ST testing was performed in food-deprived animals during seven consecutive days, with daily sessions of 15 min. During each session, forelimb function was measured as success rate (eaten pellets/total pellets), touch rate (touched pellets/total pellets) and level score (level reach/total level) for both left and right paws. A forced choice ST was performed on the 6th and 7th days of testing, assessing left and right forelimbs function respectively during these two sessions. The ST assessment was conducted 1 week after injury, in order to determine the LEV effect on forelimbs motor function after SCI.

### Histological analysis

Eight-weeks post injury, animals were deeply anesthetized by an i.p. injection of sodium pentobarbital (200 mg/ml, Eutasil/Ceva Sante Animale, Libourne, France) and transcardially perfused with 100 ml of cold 0.9% saline followed by 300 ml of 4% paraformaldehyde (PFA) in 1X phosphate-buffered saline (PBS). A rough dissection of the vertebral column and spinal cord was performed and tissues were fixed in a solution of 4% PFA for 24 h (4 °C). The spinal cord was then dissected from the vertebral column and immersed in a cryoprotectant solution −30% sucrose, for 48 h at 4 °C. Afterwards, 2 cm length of spinal cord tissues, centered on the lesion, were submerged in optimal cutting temperature (OCT) embedding medium, frozen on dry ice, and stored at −20 °C.

Regarding tissue sectioning, on the thoracic contusion model cross-sections and cervical hemisection tissue, a longitudinal-section of 20 µm was performed using a cryostat (Leica CM1900, LeicaBiosystems, Nussloch, Germany) and thaw-mounted onto charged microscope slides (Superfrost Plus, Thermo Scientific, Massachusetts, USA).

### Immunohistochemistry protocol

For immunofluorescence staining, slices were washed with PBS, permeabilized with 0.2% Triton X-100 for 10 m and blocked with 5% fetal calf serum in 0.2% Triton X-100 for 30 m. Afterwards, the following primary antibodies were incubated overnight at room temperature (RT): rabbit anti-GFAP for astrocytes (1:200; Dako Denmark, Glostrup, Danmark), mouse anti-NeuN for neurons (1:200, Millipore, Darmstadt, German) and mouse anti-βIII-tubulin for neuronal cytoskeleton (1:1000; Promega, California, USA), rabbit anti-Olig-2 for oligodendrocytes (1:500; Millipore, Darmstadt, German) and rabbit anti-Iba-1 (1:500; Wako, Osaka, Japan). On the following day, primary antibodies were probed (2 h incubation) with the appropriate Alexa 594- or Alexa 488-conjugated secondary antibodies (1:1000; Invitrogen, Paisley UK). Sections were counterstained with DAPI for 30 m (1:1000; Sigma, Saint Louis, CA, USA) and mounted with Immu-Mount® (Thermo Scientific, Waltham, MA, USA). Between steps, 5 washes with PBS (1×) were performed. For all immunofluorescence procedures, the appropriate negative controls were obtained by omission of the relevant primary antibody. Images were acquired using a confocal point-scanning microscope, Olympus FV1000. All images were analyzed using ImageJ and FIJI software.

### Immunofluorescence analysis

The immunofluorescence analysis was performed specifically according to thoracic contusion or cervical hemisection tissue as described below.

For contusion injuries, the spinal cord tissue was analyzed by collecting photomicrographs every 150 μm both rostrally and caudally from the epicenter. The epicenter region was considered the area ranging from −300 to 300 μm surrounding the lesion epicenter. The most rostral area analyzed extended from −1200 to −300 μm from the lesion epicenter and the most caudal area analyzed extended from 300 to 1200 μm from the lesion epicenter.

For cervical hemisection. the spinal cord tissue was analyzed by collecting photomicrographs every 150 μm. The spinal cord was sliced along the dorsal-ventral axis. The dorsal region was defined by the first 750 μm combined with the gray matter anatomic organization, the medial region was demarked by the range 750–1500 μm and respective gray matter organization, and the ventral region was the 1500–2250 μm range and particular gray matter organization. Photomicrographs were obtained at epicenter, rostrally and caudally to the lesion epicenter and for dorsal, medial, and ventral regions.

After obtaining micrographs through confocal microscopy, the photos were opened with the Image J software. The multi-point tool was used for cell counting. For positive area measurements, the scale was determined first and then the images were converted to 8 bits and were processed in the menu “make binary”. Finally, using the menu “analyze particles”, the software automatically calculated the areas occupied by each marker, using the dark background as contrast.

The immunofluorescence quantification in each photomicrograph was assessed by positive-cell counting (for NeuN and Olig-2) or positive staining area (for GFAP, βIII-Tubulin and Iba-1).

Data plotted in the graphs represent mean numbers (or area) per section, except NeuN staining where is represented the sum of NeuN^+^ cell by region. Finally, the GFAP-positive area was measured on the entire spinal cord slice using lower magnification. Owing to cavitation, the GFAP-positive area is presented in percentage for the total spinal cord tissue.

### Flow cytometry analysis

The inflammatory cell population present on the spinal cord tissue was characterized by flow cytometry as previously described^50^. Briefly, 1 week post injury, rats were anesthetized and transcardially perfused with 50 ml of cold NaCl 0.9%. The spinal cord was rapidly isolated from the vertebral column where 1 cm of spinal cord centered at epicenter lesion site was collected and kept in ice-cold Dulbecco’s modified Eagle medium (DMEM). Single-cell suspensions of the spinal cord were made by mechanical dissociation in DMEM with 10% heat-inactivated fetal bovine serum (FBS) and 1% antibiotic/antimycotic (Sigma, USA). Erythrocytes were depleted with ACK lysis solution. Spinal cord cell suspensions were passed through a 70 μm mesh. Myelin debris was removed through centrifugation in a 37% Percoll gradient. The cell pellet was then washed in FACS buffer (PBS, 10% BSA, 0.1% azide).

The surface cell staining was performed by incubating a cocktail of antibodies for 30 min at 4 °C. The gating strategy can be found in supplementary data (Supplementary Fig. 7). Briefly, after excluding doublets through FSC-A vs. FSC-H scatters, 7-AAD (7-AAD viability staining solution, Biolegend) negative expression was used for gating only viable cells. Then, leukocytes were gated through positive expression of CD45 (CD45—Pacific Blue, Biolegend). Infiltrative inflammatory cells were gated by high expression of CD45. Expression of CD11b/c (CD11b/c—Pe.Cy7, Biolegend) was used to gate infiltrative myeloid cells (CD45^high^CD11b/c^+^) and microglia (CD45^low^CD11b/c^+^). CD45^high^ cells but negative for CD11b/c were identified as infiltrative lymphoid cells and further gated as T or B cells through CD3 (CD3—PE, Biolegend) or CD45RA (CD45RA—APC, Biolegend) expression, respectively. After washing, cells were re-suspended in 200 μl of FACS buffer. Counting beads (Precision Count Beads, Biolegend) were added to the single cell suspensions according to manufacturer instructions to calculate final cell concentrations. Cells and beads were acquired in LSRII Flow Cytometer (BD, Pharminogen, California, USA) and analyzed with the Flow Jo software version 10.4.

### Astrocyte primary cultures

Neonatal rat cortices were isolated from P4 Wistar Han rat pups as previously described^51^. Astrocyte cell cultures were maintained at 37 °C in a 5% CO_2_ atmosphere, in Dulbecco’s modified Eagle medium (DMEM, Gibco, USA) supplemented with 10% FBS and 1% antibiotic/antimycotic (Sigma, USA). Astrocytes were plated at 1 × 10^5^ cells/cm^3^ in 24-well plates (Thermo Scientific, USA), pre-coated with poly-d-lysine (Sigma, USA), and maintained in culture for 4 days with medium changes every 2 days.

Four days after isolation, glial cell cultures were randomized into two groups: CTRL and LEV (*n* = 8) and treated with DMEM and DMEM with 1 mM of LEV. One hour after treatment, 1 mM of glutamate (Sigma, USA) was added to astrocytes. In order to determine the cells glutamate uptake, an aliquot (200 μl) of medium from each well was collected at 1, 5, 10, and 20 min and glutamate concentration was determined by HPLC as previously described and the variation of glutamate in the medium was determined (ΔGlutamate uptake = [Glu]_Final_ – [Glu]_Inicial_). Then, cells were processed for immunocytochemistry. Briefly, after fixation with PFA, cells were permeabilized and incubated with blocking solution. Samples were then incubated with the primary antibodies overnight at 4 °C: rabbit anti-GFAP for astrocytes (1:200; Dako Denmark, Glostrup, Denmark) and rabbit anti-EAAT1 for the EAAT1 (1:200; Abcam, UK). On the following day, samples were incubated with appropriate Alexa 594- or Alexa 488-conjugated secondary antibodies (1:1000; Invitrogen, Paisley UK) and DAPI.

### Statistical analysis

Statistical analysis was performed using GraphPad Prism 6.00 software. The normality of the data was evaluated by the Kolmogorov–Smirnov normality test. Data from BBB score and ST was assessed by a repeated measure ANOVA test. Differences between groups were compared with the post hoc Bonferroni test. Western blot, lipid peroxidation, HPLC, ABT, GW, MST, GS, EPT, and histological data was analyzed using the Student’s *t*-test. Statistical significance was defined for *p* < 0.05 (95% confidence level). Data is presented on text as mean ± standard deviation (SD) and on figures is shown as mean ± standard error (SEM).

### Reporting summary

Further information on research design is available in the Nature Research Reporting Summary linked to this article.

## Supplementary information

Supplementary Material

Reporting Summary Checklist

## Data Availability

The data that support the findings of this study are available from the corresponding author upon reasonable request.
