# Supplementary material for: Levetiracetam treatment leads to functional recovery after thoracic or cervical injuries of the spinal cord
Source: NPJ Regen Med. 2021 Mar 2;6:11. doi: 10.1038/s41536-021-00121-7 (PMC7977146; doi:10.1038/s41536-021-00121-7)
Supplement: Supplementary file 1 — Supplementary Material [file 41536_2021_121_MOESM1_ESM.pdf]

Levetiracetam Treatment Leads to Functional Recovery after Thoracic or Cervical Injuries of the Spinal Cord

Rui Lima, Eduardo D. Gomes, Jorge R. Cibrão, Luís A. Rocha, Rita C Assunção-Silva, Cláudia S. Rodrigues, Andreia Neves-Carvalho, Susana Monteiro, António J. Salgado, Nuno A. Silva

Supplementary Information

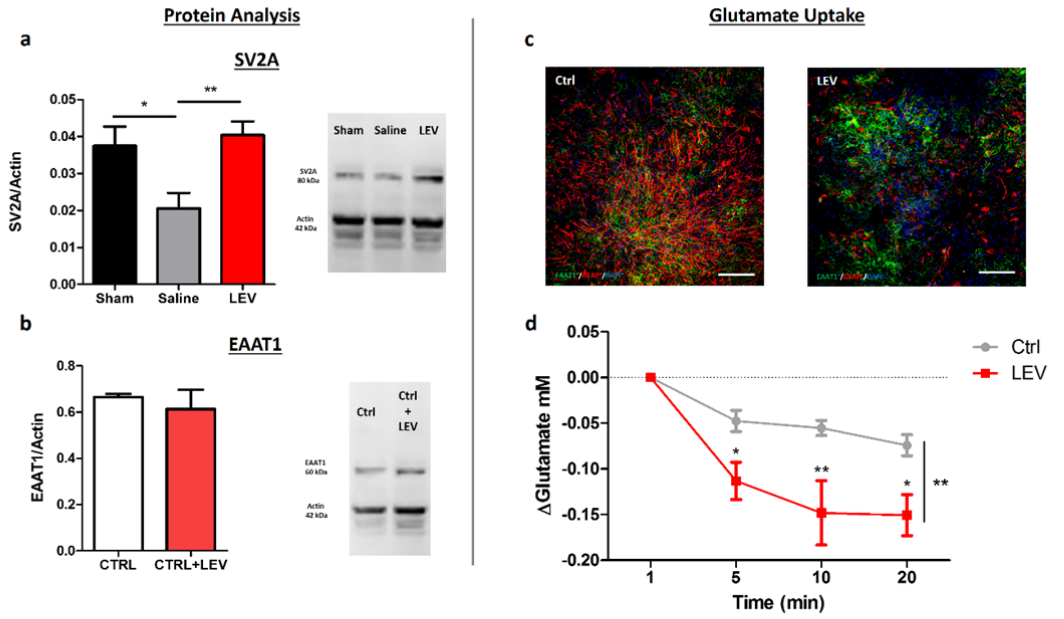

Supplementary Figure 1: LEV treatment restores the SV2A levels after SCI and increases glutamate uptake in primary astrocytes cultures. (a) SCI decreased SV2A levels and LEV administration lead to the restoration of SV2A to healthy control levels. Sham n=6; Saline n=6; LEV n=6. (b) LEV treatment do not affect EAAT1 healthy spinal cord tissue. Ctrl n=6; Ctrl+LEV n= 6. (c) Representative microphotograph of astrocytes primary cultures for saline (Ctrl) and LEV-treated cells (LEV): Red –GFAP; Green – EAAT1; Blue - DAPI. (d) LEV treated astrocytes were able to uptake significantly more glutamate from the culture medium compare to saline treated astrocytes. Saline n=8; LEV n=8 Values shown as mean ± SEM. *- $p < 0.05$; **- $p < 0.01$. Scale bar = 200 µm.

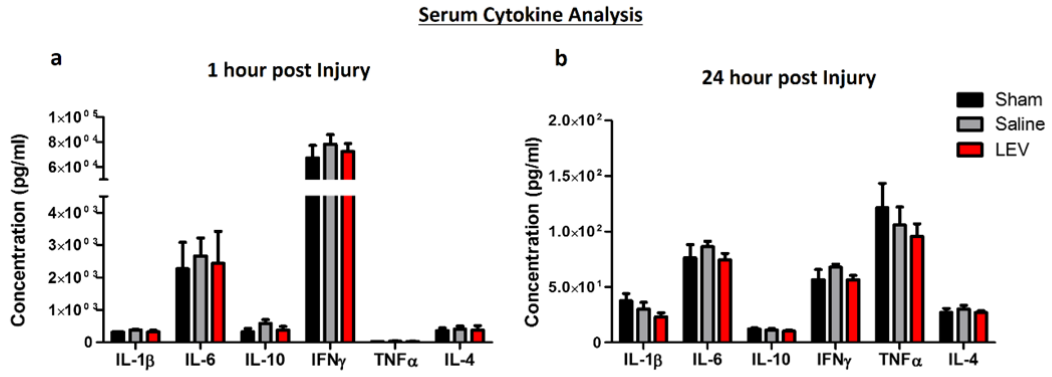

Supplementary Figure 2: Treatment does not promote changes on the acute cytokines levels in blood serum 1h and 24h after SCI. (a) Cytokine levels in the blood serum 1h after injury. (b) Cytokine levels in the blood serum 24h after SCI. Sham n=3; Saline n=6; LEV n=6. Values shown as mean ± SEM.

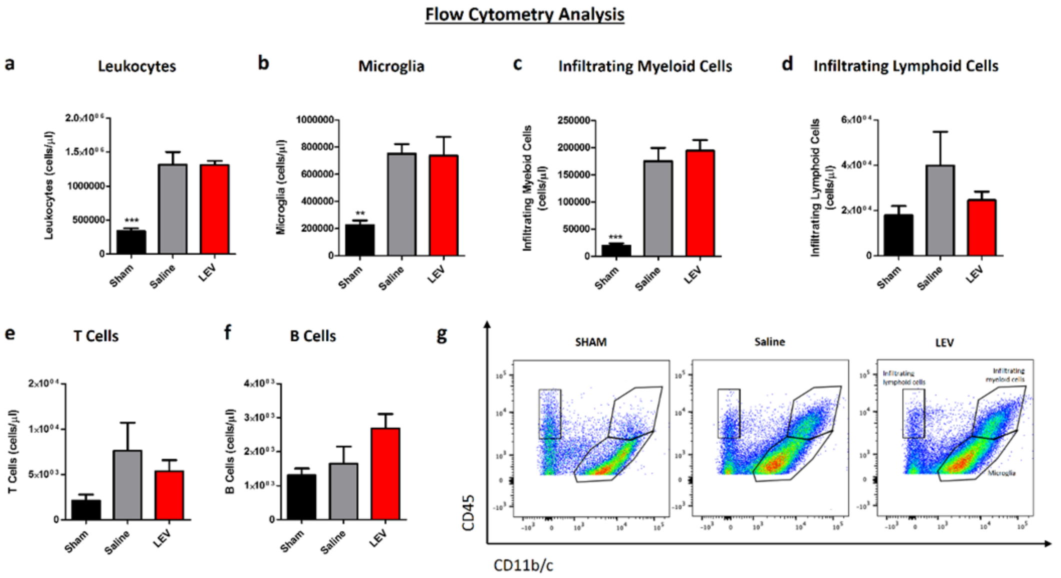

Supplementary Figure 3: LEV administration does not impact sub-acute inflammatory cell populations after SCI. One week post-injury the concentration of (a) total leukocytes, (b) microglia, (c) infiltrating myeloid cells, (d) infiltrating lymphoid cells, (e) T cells and (f) B cells in the spinal cord was analyzed by flow cytometry. Analysis did not revealed any difference between saline and LEV-treated animals. The only significant difference observed was between non-injury and the injury groups. (g) Representative scatter plots for Sham (laminectomy), Saline and LEV-treated animals. Sham n=6; Saline n=6; LEV n=6. Values shown as mean ± SEM.

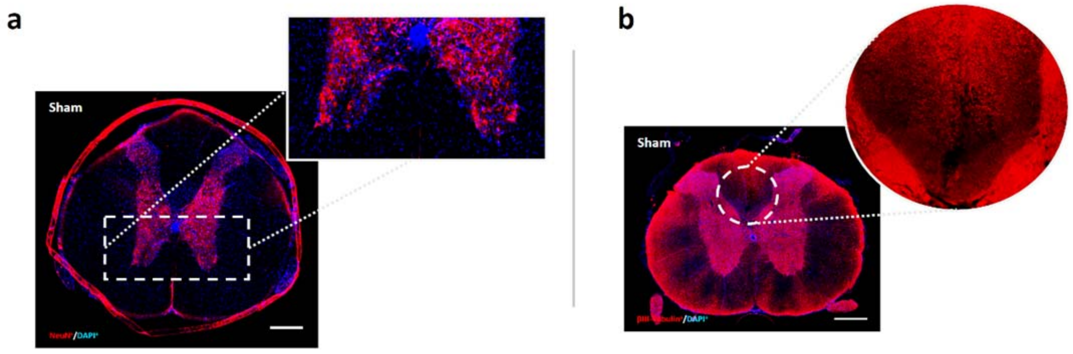

Supplementary Figure 4: Cross sections of spinal cord tissue from Sham animals stained for motor neurons and neuronal fibers at the cortical spinal tract. (a) Representative confocal photomicrographs of healthy thoracic spinal cord tissue staining $NeuN^+$. (b) Representative confocal photomicrographs of βIII-tubulin+ fibers at the CST in healthy spinal cord tissue. Scale bar = 200 µm.

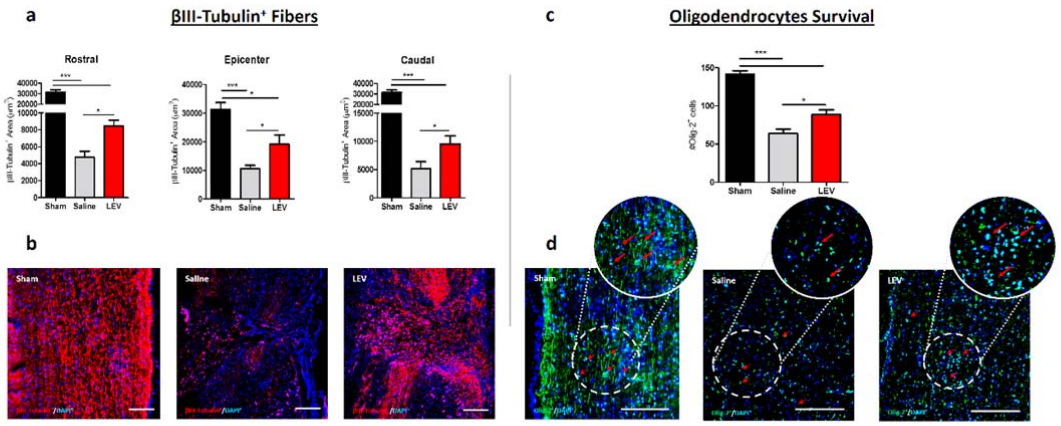

Supplementary Figure 5: LEV treatment promoted protection of axons and oligodendrocytes on cervical SCI animals. (a) The neuronal fibers positive area significantly increased along rostral, epicenter and caudal regions on LEV treated animals. (b) Representative confocal photomicrographs of βIII-tubulin+ fibers at the epicenter region. (c) Olig-2+ quantification shown that LEV treated animals presented higher number of oligodendrocytes when compared to the saline treatment group. (d) Representative confocal photomicrographs of Olig-2+ cells near to the epicenter region. Sham n=3; Saline n=6; LEV n=7. Values shown as mean ± SEM. *- $p < 0.05$; **- $p < 0.01$; ***- $p < 0.001$. Scale bar = 200 µm.

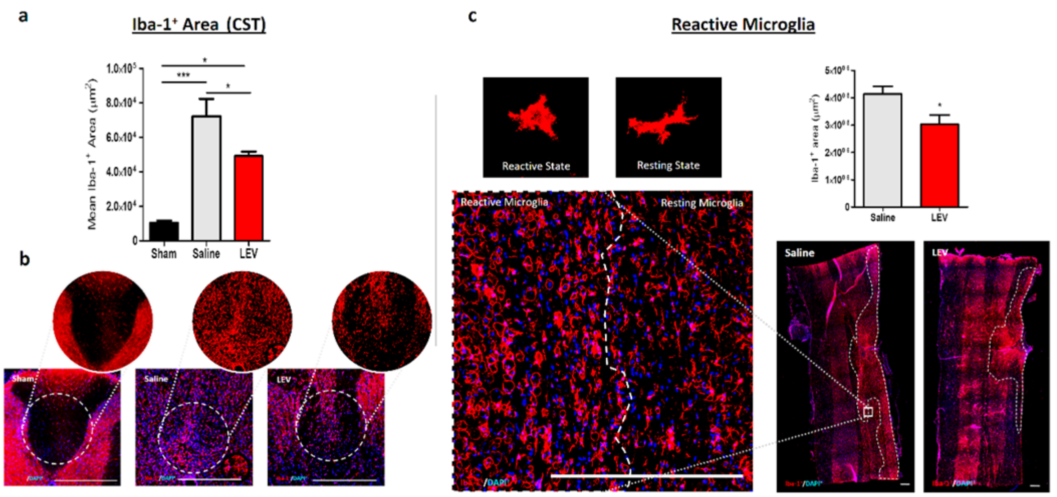

Supplementary Figure 6: LEV acute treatment reduced microglia infiltration on both thoracic and cervical SCI animals. (a) The microglia cell positive area at corticospinal tract was decreased on LEV treated animals. (b) Representative confocal photomicrographs of Iba-1+ cells at corticospinal tract. Saline n=8; LEV n= 14. (c) Iba-1+ quantification showed that LEV treated animals presented a smaller microglia reactive distribution area when compared to saline treatment group. Reactive microglia was not found on sham animals. Representative confocal photomicrographs of Iba+ staining along the spinal cord. Sham n=3; Saline n=6; LEV n=7. Values shown as mean ± SEM. *- $p < 0.05$; ***- $p < 0.001$. Scale bar = 400 µm.

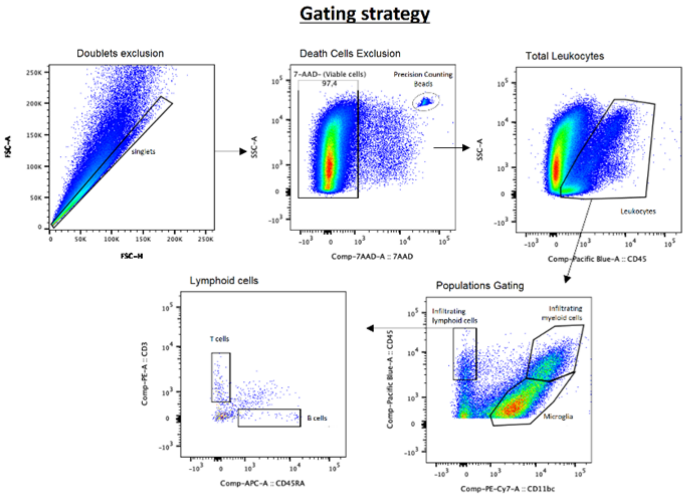

Supplementary Figure 7: Gating strategy used for flow cytometry analysis of spinal cord single-cell suspensions. Doublets were excluded by FSC-A vs FSC-H scatter. Live leukocytes were gated after

excluding 7-AAD+ cells and selecting CD45+ cells. Infiltrative leukocytes were selected by high expression of CD45 and microglia by low expression of CD45. Infiltrative myeloid cells were selected by positive expression of CD11b/c and infiltrative lymphoid cells by negative expression. Within the infiltrative lymphoid populations, T cells were selected by positive CD3 expression and B cells by positive CD45RA expression.
