# Supplementary material for: Levetiracetam treatment leads to functional recovery after thoracic or cervical injuries of the spinal cord
Source: NPJ Regen Med. 2021 Mar 2;6:11. doi: 10.1038/s41536-021-00121-7 (PMC7977146; doi:10.1038/s41536-021-00121-7)
Supplement: Supplementary file 2 — Reporting Summary Checklist [file 41536_2021_121_MOESM2_ESM.pdf]

nature research

Corresponding author(s): Nuno A. Silva

Last updated by author(s): Oct 2, 2020

Reporting Summary

Nature Research wishes to improve the reproducibility of the work that we publish. This form provides structure for consistency and transparency in reporting. For further information on Nature Research policies, see our Editorial Policies and the Editorial Policy Checklist.

Statistics

For all statistical analyses, confirm that the following items are present in the figure legend, table legend, main text, or Methods section.

n/a	Confirmed	
☐	☒	The exact sample size (*n*) for each experimental group/condition, given as a discrete number and unit of measurement
☐	☒	A statement on whether measurements were taken from distinct samples or whether the same sample was measured repeatedly
☐	☒	The statistical test(s) used AND whether they are one- or two-sided<br>*Only common tests should be described solely by name; describe more complex techniques in the Methods section.*
☒	☐	A description of all covariates tested
☐	☒	A description of any assumptions or corrections, such as tests of normality and adjustment for multiple comparisons
☐	☒	A full description of the statistical parameters including central tendency (e.g. means) or other basic estimates (e.g. regression coefficient) AND variation (e.g. standard deviation) or associated estimates of uncertainty (e.g. confidence intervals)
☐	☒	For null hypothesis testing, the test statistic (e.g. *F*, *t*, *r*) with confidence intervals, effect sizes, degrees of freedom and *P* value noted<br>*Give P values as exact values whenever suitable.*
☒	☐	For Bayesian analysis, information on the choice of priors and Markov chain Monte Carlo settings
☒	☐	For hierarchical and complex designs, identification of the appropriate level for tests and full reporting of outcomes
☒	☐	Estimates of effect sizes (e.g. Cohen's *d*, Pearson's *r*), indicating how they were calculated

Our web collection on statistics for biologists contains articles on many of the points above.

Software and code

Policy information about availability of computer code

Data collection	Flow Cytometry:LSRII Flow Cytometer;Flow Jo software version 10.4 (BD, Pharminogen, California, USA)<br>Western Blot: Image-Lab software<br>Serum Cytokines: MAGPIX Luminex's xMAP® instrument (Luminex, Austin, TX, USA)<br>Microplate reader: Infinite® M200 NanoQuant microplate reader (Tecan, Switzerland).<br>HPLC: Gilson Uniprot Software version 5.11
Data analysis	GraphPad Prism 6.00 software

For manuscripts utilizing custom algorithms or software that are central to the research but not yet described in published literature, software must be made available to editors and reviewers. We strongly encourage code deposition in a community repository (e.g. GitHub). See the Nature Research guidelines for submitting code & software for further information.

Data

Policy information about availability of data

All manuscripts must include a data availability statement. This statement should provide the following information, where applicable:

- Accession codes, unique identifiers, or web links for publicly available datasets
- A list of figures that have associated raw data
- A description of any restrictions on data availability

The data that support the findings of this study are available from the corresponding author upon reasonable request.

Field-specific reporting

Please select the one below that is the best fit for your research. If you are not sure, read the appropriate sections before making your selection.

☒ Life sciences ☐ Behavioural & social sciences ☐ Ecological, evolutionary & environmental sciences

For a reference copy of the document with all sections, see nature.com/documents/nr-reporting-summary-flat.pdf

Life sciences study design

All studies must disclose on these points even when the disclosure is negative.

Sample size	The number of animals on this study was determine by Gpower software (University of Kiel, Germany) with results obtain from a pilot study with n=5 animals per group.
Data exclusions	During data analysis an exclusion criteria was established for both behavioral and histological assessments. Animals that did not present a BBB of zero three days after injury were excluded of the behavioral and histological analysis. Spinal cord sections that were shattered, cracked, folded or sections washed off during immunostaining procedure were excluded from analysis.
Replication	All procedures where replicated twice.
Randomization	Animals were randomized to treatment and all data collection
Blinding	All data collection was obtained blinded to the treatment group

Reporting for specific materials, systems and methods

We require information from authors about some types of materials, experimental systems and methods used in many studies. Here, indicate whether each material, system or method listed is relevant to your study. If you are not sure if a list item applies to your research, read the appropriate section before selecting a response.

Materials & experimental systems

n/a	Involved in the study	
☐	☒	Antibodies
☒	☐	Eukaryotic cell lines
☒	☐	Palaeontology and archaeology
☐	☒	Animals and other organisms
☒	☐	Human research participants
☒	☐	Clinical data
☒	☐	Dual use research of concern

Methods

n/a	Involved in the study	
☒	☐	ChIP-seq
☐	☒	Flow cytometry
☒	☐	MRI-based neuroimaging

Antibodies

Antibodies used

Anti-SV2A: Rabbit polyclonal to SV2A, Abcam, ab32942, Lot. GR277874-1
Anti-EAAT1: Rabbit polyclonal to EAAT1, Abcam, ab416, Lot.GR285370-1
Anti-actin: Mouse monoclonal to beta-actin, Abcam, ab8224, Lot.GR221876-7
Anti-GFAP:Rabbit polyclonal to GFAP, Dako, Z0334, Lot.20002902
Anti-NeuN: Mouse monoclonal to NeuN, Millipore, MAB377, Lot.3167020
Anti-B3 tubulin: Mouse monoclonal to BIII tubulin, Promega, G7121, Lot.0000346917
Anti-Olig2: Rabbit polyclonal to Olig2, Millipore, ABN899, Lot.3083557
Anti-Iba1: Rabbit anti-iba1, Wako, 019-19741, Lot.PTE0555
Pacific Blue™ anti-rat CD45, OX-1, cat: 202225, Biolegend, Lot.B273075
PE/Cyanine7 anti-rat CD11b/c , OX-42, cat:201817, Biolegend, Lot.B260582
PE anti-rat CD3, 1F4, cat:201411, Biolegend, Lot.B215290
APC anti-rat CD45RA, OX-33, cat:202313, Biolegend, Lot.B248447

Validation

According Manufacturer:

Anti-SV2A: Reactivity: Mouse, Rat, Human; Application: WB, IP
Anti-EAAT1: Reactivity: Rat, Human; Application: WB, IHC, ICC
Anti-actin: Reactivity:Mouse, Rat, Rabbit, Chicken, Cow, Cat, Dog, Human, Pig, Xenopus laevis, Drosophila melanogaster, Schizosaccharomyces pombe, Chinese hamster ; Application: FC, WB, IHC
Anti-GFAP: Reactivity: Marine lamprey, Human, Mouse, Rat, Dogs, Chicken, Zebrafish, Sheep; Application: WB, IHC, ICC
Anti-NeuN: Reactivity: Avian, Chicken, Ferret, Human, Mouse, Pig, Rat, Salamander; Application: FC, ICC, IHC, IF, IP, WB
Anti-B3 tubulin: Reactivity: Most mammalian species; Application: WB, ICC, IHC

Anti-Olig2: Reactivity: Human, Mouse, Rat; Application: WB, ICC, IHC
Anti-Iba1: Reactivity: Human, Mouse, Rat; Application: ICC, IHC
Pacific Blue™ anti-rat CD45: Reactivity:rat; Application FC - quality tested
PE/Cyanine7 anti-rat CD11b/c; Reactivity:rat; Application FC - quality tested
PE anti-rat CD3, Reactivity:rat; Application FC - quality tested
APC anti-rat CD45RA, Reactivity:rat; Application FC - quality tested

ICC - Immunocytochemistry; IHC - Immunohistochemistry; IF - Immunofluorescence; WB - Western Blot; FC - Flow Cytometry; IP- Imunoprecipitation

Animals and other organisms

Policy information about studies involving animals; ARRIVE guidelines recommended for reporting animal research

Laboratory animals	Female Wistar Han Rat 10 weeks aged
Wild animals	The study did not involve wild animals
Field-collected samples	The study did not involve samples collected from the field
Ethics oversight	All procedures were carried out in accordance to EU directive 2010/63/EU and were approved by the ethical committee in life and health sciences (ID: SECVS116/2016, University of Minho, Braga, Portugal).

Note that full information on the approval of the study protocol must also be provided in the manuscript.

Flow Cytometry

Plots

Confirm that:

☒ The axis labels state the marker and fluorochrome used (e.g. CD4-FITC).

☒ The axis scales are clearly visible. Include numbers along axes only for bottom left plot of group (a 'group' is an analysis of identical markers).

☒ All plots are contour plots with outliers or pseudocolor plots.

☒ A numerical value for number of cells or percentage (with statistics) is provided.

Methodology

Sample preparation	Briefly, one week post injury, rats were anesthetized and transcardially perfused with 50 ml of cold NaCl 0.9 %. The spinal cord was rapidly isolated from the vertebral column where 1 cm of spinal cord centered at epicenter lesion site was collected and kept in ice-cold DMEM. Single-cell suspensions of the spinal cord were made by mechanical dissociation in DMEM with 10% heat-inactivated FBS and 1% antibiotic/antimycotic (Sigma, USA). Erythrocytes were depleted with ACK lysis solution. Spinal cord cell suspensions were passed through a 70 µm mesh. Myelin debris was removed through centrifugation in a 37% Percoll gradient. The cell pellet was then washed in FACS buffer (PBS, 10% BSA, 0.1 % azide).
Instrument	LSRII Flow Cytometer (BD, Pharminogen, California, USA)
Software	Flow Jo software version 10.4
Cell population abundance	No post-sort fractioning was used. Total single-cell suspensions from 1cm of the spinal cord centered at the epicenter lesion was used. Myelin was removed using Percoll 37% gradient as described in the methods section.
Gating strategy	After excluding doublets through FSC-A vs FSC-H scatters, 7-AAD (7-AAD viability staining solution, Biolegend) negative expression was used for gating only viable cells. Then leukocytes were gated through positive expression of CD45 (CD45 - Pacific Blue, Biolegend). Infiltrative inflammatory cells were gated by high expression of CD45. Expression of CD11b/c (CD11b/c – Pe.Cy7, Biolegend) was used to gate infiltrative myeloid cells (CD45highCD11b/c+) and microglia (CD45lowCD11b/c+). CD45high cells but negative for CD11b/c were identified as infiltrative lymphoid cells and further gated as T or B cells through CD3 (CD3 – PE, Biolegend) or CD45RA (CD45RA – APC, Biolegend) expression, respectively.

☒ Tick this box to confirm that a figure exemplifying the gating strategy is provided in the Supplementary Information.
